# Data Triangulation in Consumer Neuroscience: Integrating Functional Neuroimaging With Meta-Analyses, Psychometrics, and Behavioral Data

**DOI:** 10.3389/fpsyg.2020.550204

**Published:** 2020-11-05

**Authors:** C. Clark Cao, Martin Reimann

**Affiliations:** ^1^Department of Marketing and International Business, Lingnan University, Tuen Mun, Hong Kong; ^2^Department of Marketing, University of Arizona, Tucson, AZ, United States

**Keywords:** consumer neuroscience, fMRI, data triangulation, neuroimaging database, validity

## Abstract

This article reviews a wide range of functional magnetic resonance imaging (fMRI) studies conducted in the field of consumer neuroscience to (1) highlight common interpretative approaches of neuroimaging data (i.e., forward inference and reverse inference), (2) discuss potential interpretative issues associated with these approaches, and (3) provide a framework that employs a multi-method approach aimed to possibly raise the explanatory power and, thus, the validity of functional neuroimaging research in consumer neuroscience. Based on this framework, we argue that the validity of fMRI studies can be improved by the triangulation of (1) careful design of neuroimaging studies and analyses of data, (2) meta-analyses, and (3) the integration of psychometric and behavioral data with neuroimaging data. Guidelines on when and how to employ triangulation methods on neuroimaging data are included. Moreover, we also included discussions on practices and research directions that validate fMRI studies in consumer neuroscience beyond data triangulation.

## Introduction

Since the dawn of consumer neuroscience, researchers have employed neuroimaging techniques, especially functional magnetic resonance imaging (fMRI), to explore latent mental processes underlying consumer behavior (e.g., Yoon et al., 2006; Hedgcock and Rao, 2009; Reimann et al., 2010; Meade and Craig, 2012; Plassmann and Weber, 2015). Thanks to their collective effort through more than a decade of research, consumer neuroscientists have accumulated a wealth of insights that sheds light upon many aspects of consumer behavior. However, an unresolved challenge exists: that of reverse inference. Defined as the use of neuroimaging data to infer the presence of specific cognitive and affective processes (Poldrack, 2008), reverse inference may be tricky when interpretating neuroimaging results (Reimann et al., 2011, 2016; Plassmann et al., 2012, 2015).

In this article, we attempt to summarize potential issues associated with reverse inference, and we argue that proper data triangulation can help address these issues. Herein, we define data triangulation as the practice of utilizing multiple methods and data sources to study the same phenomenon (Patton, 1999; Carter et al., 2014). In neuroimaging research, triangulation would thus entail integrating neuroimaging data with data from meta-analyses, psychometric assessments, and consumer behavior.

Because the neuroimaging techniques employed in consumer neuroscience often consist of fMRI, we focus exclusively on fMRI research in our review of research papers, examinations of research methods, and recommendations to consumer researchers (hereinafter, we use the terms “neuroimaging” and “fMRI” synonymously). However, many of our analyses and recommendations could possibly be applied to other imaging techniques as well.

This paper is organized as follows. First, we review more than 50 empirical articles employing fMRI techniques published in consumer and marketing research journals as well as neuroscience journals, and we use this review to discusses fMRI’s contributions to consumer research. Second, we discuss two major interpretative approaches of neuroimaging research—forward inference and reverse inference—and explain how these approaches contribute to understanding consumer behavior. Third, we discuss potential issues and challenges associated with reverse inference. Fourth, we argue that the utilization of data triangulation of fMRI data with meta-analytic data as well as psychometric and behavioral data can enhance the validity of fMRI studies. Fifth, we provide and discuss several recommendations for future consumer neuroscience research.

## Functional Neuroimaging in Consumer Research

Over the past one and a half decades, the field of consumer neuroscience has significantly contributed to consumer research. In Table 1, we have reviewed and attempted to summarize more than 50 empirical articles utilizing fMRI techniques. The works summarized here have been published in consumer research journals such as the *Journal of the Academy of Marketing Science*, the *Journal of Consumer Research*, the *Journal of Marketing Research*, the *Journal of Consumer Psychology*, and the *Journal of the Association for Consumer Research*, as well as journals in fields outside consumer research, including psychology, neuroscience, and the general science. For journals outside consumer research, we have focused our review on *Cortex*, *Journal of Neuroscience*, *Journal of Neuroscience, Psychology, and Economics, NeuroImage*, *Neuron*, *Psychological Science*, the *Proceedings of the National Academy of Sciences*, and *Social Cognitive and Affective Neuroscience*, in which some of the earlier work on consumer neuroscience appeared and more recent work has continued to appear. For consumer research journals, the list of included works is based on research results for methodological keywords such as “neuroimaging” and “fMRI.” For journals outside consumer research, the list of included works is focused on fMRI studies generated from searching keywords such as “consumer neuroscience,” “neuromarketing,” “market,” “marketing,” “consumer,” and “purchase.” Given our focus on specific journals and keywords, we acknowledge that some works related to consumer neuroscience may not be included in this review. Additionally, since our article’s focus is on fMRI, we would like to note that important work has been done using other imaging techniques such as electroencephalography (EEG). Nevertheless, the works included in Table 1 cover a broad range of topics relevant to consumer researchers, including theoretical domains such as information processing (Craig et al., 2012; Esch et al., 2012), judgment and decision making (Hedgcock and Rao, 2009; Karmarkar et al., 2015), and goals and motivation (Hedgcock et al., 2012; Wiggin et al., 2019), as well as substantive domains such as advertising (Craig et al., 2012; Venkatraman et al., 2012; Falk et al., 2016), pricing (Plassmann et al., 2008; Plassmann and Weber, 2015), branding (Reimann et al., 2012, 2018; Chan et al., 2018), and packaging design (Reimann et al., 2010). The table also examines the papers’ usage of meta-analyses as well as psychometrics/behavioral data, two major data source that we recommend triangulating with the fMRI data. Taken together, Table 1 provides a summary of the status of the consumer neuroscience literature and the type of data being used, showing that over the past one and a half decades researchers have started to integrate different data forms in their investigations.

**TABLE 1:** Consumer neuroscience research in marketing, consumer research, and neuroscience journals (chronological order).

Author(s) and Journal*	**fMRI-related research question**	**Major findings**	**Identified brain areas**	**Role of fMRI in study**	**Meta-analysis**	**Triangulation with psychometric and/or behavioral data**
McClure et al. (2004), *Neuron*	What are the neural correlates of the consumer preference for Coke and Pepsi two similar drinks, and how can brand knowledge influence the behavioral preference and brain responses.	When the brands are delivered anonymously, brain activity in the ventromedial prefrontal cortex correlates with participant’s behavioral preferences for the drinks. However, when participants know that it is Coke that they are drinking, behavioral preferences are drastically influenced and activity in the hippocampus, dorsolateral prefrontal cortex, and midbrain are also found.	•Ventromedial prefrontal cortex (reward). •Dorsolateral prefrontal cortex, hippocampus, and midbrain (recall of cultural influences).	Investigate neural correlates		Yes
Yoon et al. (2006) *JCR*	Whether semantic judgments about products and persons are processed similarly.	Mental judgments about persons and products are processed differently.	•Medial prefrontal cortex (semantic judgments about persons). •Left inferior prefrontal cortex (semantic judgments about products).	Distinguish multiple processes		
Knutson et al. (2007), *Neuron*	How product preference and price are captured in consumer brain and how their neural correlates predict purchase.	Product preference activates the nucleus accumbens. Excessive price, on the other hand, activates the insula and deactivates the medial prefrontal cortex. Activities in each of these regions can independently predict purchase.	•Nucleus accumbens (gain prediction). •Insula (loss prediction). •Medial prefrontal cortex (gain prediction errors).	Investigate neural correlates and predict behaviors		
Plassmann et al. (2007), *JN*	How willingness to pay is encoded in the brain.	Willingness to pay is encoded in the medial orbitofrontal cortex and in the dorsolateral prefrontal cortex.	Medial orbitofrontal cortex (reward and valuation)	Investigate neural correlates		
Weber et al. (2007), *NI*	What are the neural correlates of reference-dependence of utility evaluation (i.e., the endowment effect), before and after the buying/selling decision in real market contexts.	Fear-related processes are observed when a product is being sold but not when it is being bought. After the decision is made, reward related processes are observed if a high external reference price of the product is provided.	•Left amygdala (fear and loss). •Caudate nucleus (action planning and monetary rewards expectation). •Orbitofrontal cortex (reward). •Anterior cingulate (mental conflicts, reward, and valuation).	Investigate neural correlates		
Bray et al. (2008), *JN*	What are the neural correlates of the outcome-specific transfer (the consumer tendency to choose the option with a particular outcome when a Pavlovian cue is present, if the Pavlovian cue has previously been associated with that outcome).	A region in the ventral caudolateral putamen is associated with outcome-specific transfer.	Ventral putamen (reward outcome and reward learning)	Investigate neural correlates		
Klucharev et al. (2008), *SCAN*	What are the neural correlates of the persuasive effect of high expertise of the communicator (i.e., the “expert power”)	Expert content is associated with brain regions associated with active semantic elaboration, memory formation, and trustful behavior, reward processing, and learning	•Left dorsomedial prefrontal cortex, anterior cingulate, superior temporal sulcus (semantic processing). •Hippocampus and parahippocampal gyrus (memory). •Caudate nucleus (trust, reward, learning).	Investigate neural correlates		
Plassmann et al. (2008), *PNAS*	How the price of a product influences the neural computations with its experienced pleasantness.	Increase of the price of a wine increases both the activation in the medial orbitofrontal cortex and the subjective report of pleasantness.	•Medial orbitofrontal cortex (encoding of experienced pleasantness).	Identify psychological processes		
De Martino et al. (2009), *JN*	Whether reference-independent and -dependent value computations are reflected in the same region of the brain.	The computation of reference-independent value is associated with the activity in the orbitofrontal cortex and dorsal striatum. On the other hand, the computation of reference-dependent value is associated with the activity in the ventral striatum.	•Orbitofrontal cortex. •Dorsal striatum. •Ventral striatum.	Investigate neural correlates		
Dietvorst et al. (2009), *JMR*	How to judge salespersons’ ability of interpersonal-mentalizing (i.e., inferring the viewpoints of customers).	Interpersonal mentalizing process (“read” others’ minds, process subtle interpersonal cues, and then adjust volitions accordingly).	Medial prefrontal cortex and bilateral temporo-parietal junctions (interpersonal-mentalizing skills)	Predict behaviors		Yes
Hedgcock and Rao (2009), *JMR*	Why the introduction of a decoy option (i.e., an option that is dominated and thus should not the influence preference for other options) into the choice set makes one of the original options more attractive (the “attraction effect”).	The decoy option produces the attraction effect by reducing negative emotions experienced by the decision maker.	•Amygdala (negative emotions). •Medial prefrontal cortex (self-referential evaluation of preferences). •Dorsal lateral prefrontal cortex (use of decision rules). •Anterior cingulate cortex (conflict monitoring). •Right inferior parietal lobule (numerical magnitude processing).	Identify psychological processes		Yes
Berns et al. (2010), *NI*	How the popularity of music influences individual rating thereof.	Popularity rating influences individual rating by creating anxiety due to a mismatch between one’s own and others’ preferences. This anxiety in turn drives choice switch in the direction of popularity rating. Popularity does not seem to change the individual preferences for music *per se*.	•Caudate nucleus (reward and likability). •Anterior insula and anterior cingulate (physiological arousal and negative affective states). •Middle temporal gyrus (semantic processing).	Identify psychological processes, and test competing theories		
Linder et al. (2010), *NI*	How consumers evaluate food with (vs. without) organic labels.	Organic food label is associated with increased activity in the ventral striatum. The difference in such activity can predict the real-world consumption behavior of organic food.	Ventral striatum (reward)	Identify psychological processes and predict behavior		
Rademacher et al. (2010), *NI*	Whether the anticipation and consumption of rewards (monetary and social) involves the same or different neural networks.	Anticipation and consumption of rewards involve dissociable neural networks. Anticipation of both monetary and social reward involves the reward system including the ventral striatum. On the other hand, the consumption of monetary reward involves the thalamus, whereas the consumption of social reward involves the amygdala.	•Brain reward system including ventral striatum (reward anticipation). •Amygdala (social reward consumption). •Thalamus (monetary reward consumption).	Investigate neural correlates		
Reimann et al. (2010), *JCP*	How consumers process aesthetic package design.	The reward system in the brain plays a role in processing aesthetic package design.	•Striatum (anticipated reward). •Ventromedial prefrontal cortex (reward outcome).	Identify neural correlates and psychological processes		Yes
Schaefer and Rotte (2010), *SCAN*	How brands are represented in the brain.	Brands rated higher on “social competence” is associated with greater activation in the medial prefrontal cortex, whereas brands rated higher on “potency” is associated with greater activation in the superior frontal gyri.	•Medial prefrontal cortex (social cognition). •Superior frontal gyri (working memory).	Investigate neural correlates		
Smith et al. (2010), *JN*	Whether expected and experienced value are reflected in the same part of the brain.	Experienced value is reflected in the anterior ventromedial prefrontal cortex, whereas expected value is reflected in the posterior ventromedial prefrontal cortex.	Ventromedial prefrontal cortex (reward).	Investigate neural correlates		
Tusche et al. (2010), *JN*	What brain regions can predict consumer choice, and how product attention influences such predictions.	Insula and medial prefrontal cortex activation patterns can predict consumer choice, both under high and low product attention.	Insula and medial prefrontal cortex (reward).	Predict behavior		
Kang et al. (2011), *JN*	Whether hypothetical and real decision-making processes undergo the same type of valuation and choice computations or not.	Common areas in the orbitofrontal cortex and the ventral striatum are related to both hypothetical and real evaluation of goods. The difference found between the two types of decision-making are quantitative instead of qualitative.	Orbitofrontal cortex and ventral striatum, anterior cingulate cortex, caudate, inferior frontal gyrus (reward).	Identify psychological processes, and test competing theories		
Levy et al. (2011), *JN*	Does a single neural mechanism capture the reward values of options and thus predict consumer preferences, both when a decision requires subsequent choice and when the choice is absent.	The striatum and medial prefrontal cortex represent values whether choice is required or not.	Striatum and medial prefrontal cortex (reward).	Identify psychological processes and predict behavior		
Bagozzi et al. (2012), *JAMS*	What is the neural basis of customer orientation (as opposed to sales orientation) in salespersons.	Customer oriented, but not sales oriented, salespersons features greater activity in their mirror neuron systems, as well as empathy-related neural process.	•Posterior inferior frontal cortex, Broca’s area, and anterior inferior parietal lobule (mirror neuron system). •Medial prefrontal cortex, precuneus, and right inferior parietal cortex (empathy). •Insula and amygdala (empathic concern). •Inferior parietal lobule (self-other monitoring).	Investigate neural correlates		Yes
Berns and Moore (2012), *JCP*	Whether brain activation acquired from a small sample can predict product acceptance in a broader population.	Although subjective likability of the songs was not predictive of sales, activity within the ventral striatum was significantly correlated with the number of units sold.	Precuneus, orbitofrontal cortex, and ventral striatum/nucleus accumbens (reward).	Predict behavior		Yes
Craig et al. (2012), *JMR*	How consumers process deceptive advertisement claims.	Consumers process deceptive claims in two distinct stages: First, greater attention may be allocated to more deceptive information; second, more attention and belief reasoning may then be directed toward believable and moderately information.	•Amygdala, precuneus (expectation violation observed in person-to-person cheater detection). •Superior temporal and inferior parietal areas (including superior temporal sulcus and temporo-parietal junctions) (verification and assessment of claims).	Identify psychological processes and distinguish multiple processes		Yes
Esch et al. (2012), *JCP*	What are the consumer linguistic encoding/retrieval processes and how do they use declarative/experiential information in the evaluation of unfamiliar vs. familiar (strong and weak) brands.	The evaluation of unfamiliar brands is associated with linguistic encoding, whereas the evaluation of strong brands is associated with information retrieval. Moreover, consumers use experienced emotions rather than declarative information to evaluate brands.	•Broca’s area (linguistic processing). •Wernicke’s area (brand retrieval). •Pallidum (positive emotions).	Investigate neural correlates and identify psychological processes		
Falk et al. (2012), *PS*	Can neural responses collected from a small group of people predict the effectiveness of an anti-smoking television campaign.	Activations in the medial prefrontal cortex predicts the population-level effectiveness of the campaign.	Medial prefrontal cortex (individual behavior change).	Predict behavior		Yes
Hedgcock et al. (2012), *JCP*	How depletion causes self-control failure in a two-stage model (stage 1: recognizing the need for self-control; stage 2: implementing controlled responses). Specifically, whether depletion undermines stage 1 only, stage 2 only, or both.	Depletion affects the implementation stage only and leaves intact the ability to recognize the need for self-control.	•Anterior cingulate cortex (goal conflict identification). •Dorsolateral prefrontal cortex (controlled actions implementation).	Identify psychological processes, and test competing theories		Yes
Lawrence et al. (2012), *NI*	How the nucleus accumbens is associated with food cue and predicts subsequent food consumption and body mass index.	Food cue related activations in the nucleus accumbens predicts subsequent snack food consumption (but not subjective hunger) in female participants. Nucleus accumbens activities also predict body mass index but only in participants with lowered self-control.	•Left nucleus accumbens (food motivation, reward, wanting). •Ventromedial prefrontal cortex (subject hunger/appetite).	Investigate neural correlates and predict behavior		
Reimann et al. (2012), *JCP*	•How consumers relate to their beloved brands.	Emotional arousal decreases over the brand relationship span, while inclusion of the brand into the self increases over time.	Insula (urging, addiction, loss aversion, and interpersonal love)	Identify neural correlates and psychological processes		Yes
Grabenhorst et al. (2013), *NI*	How sample food labels influence consumer healthy food choice.	Food labels can bias food evaluations in the amygdala, and the strength of such biases can predict behaviors toward healthier food choices.	Amygdala (emotion).	Identify psychological processes		
Bruce et al. (2014a), *SCAN*	How children’s brains react to food brand logos in children.	Food logos (vs. baseline) are associated with activity in the orbitofrontal cortex and inferior prefrontal cortex. Food logos (vs. non-food logos) are associated with activity in the posterior cingulate cortex.	•Orbitofrontal cortex (motivation). •Inferior prefrontal cortex (cognitive control). •Posterior cingulate cortex (visual engagement).	Investigate neural correlates		
Bruce et al. (2014b), *JNPE*	How consumers react to food attributes presented to them, especially the food’s price and technology used to produce it.	Increased number of presented attributes is associated with increased activity in the dorsolateral prefrontal cortex	Dorsolateral prefrontal cortex (working memory and uncertainty).	Investigate neural correlates		
Gearhardt et al. (2014), *SCAN*	What the neural responses of food-related (vs. non-food-related) commercials, and how weight influences such neural responses.	Adolescents exhibit overall greater activation in brain regions associated with visual processing, attention, cognitive processing, movement, somatosensory responses, and reward, when they process food-related (vs. non-food-related) commercials. Moreover, during food relative to non-food commercials, obese participants feature less activation in brain regions associated with visual processing, attention, reward, and salience detection. Obese participants also feature more activation in brain regions associated with semantic control.	•Occipital gyrus, cuneus (visual processing). •Parietal lobes, posterior cerebellar lobe, temporal gyrus (attention and cognition). •Anterior cerebellar cortex (movement). •Postcentral gyrus (somatosensory response). •Orbitofrontal cortex, anterior cingulate cortex, ventromedial prefrontal cortex (reward). •Precuneus (salience detection). •Medial temporal gyrus (semantic control).	Investigate neural correlates		
Kanayet et al. (2014), *PS*	Whether numeric magnitude and monetary value are processed by distinct regions in the neural network involved in the valuation of monetary reward.	Changes in numeric magnitude is associated with activities in intraparietal sulcus, whereas monetary value is associated in the activity in orbitofrontal cortex.	•Intraparietal sulcus (numeric information). •Orbitofrontal cortex (reward, valuation).	Investigate neural correlates and distinguish multiple processes		
Lighthall et al. (2014), *JN*	How older adults compensate their age-related memory decline during memory-dependent decision-making in consumption.	Medial prefrontal cortex provides functional compensation for aging adults in memory-dependent decision-making tasks.	Medial prefrontal cortex and its connectivity with dorsolateral prefrontal cortex.	Identify psychological processes		
Tang et al. (2014), *PS*	How actual and perceived foods’ caloric content influences the neural predictors of food choice.	Actual, but not perceived, food caloric content is associated with activations in the ventromedial prefrontal cortex, which is known as a predictor of food choice. The ventromedial prefrontal cortex demonstrates a functional connectivity with an appetitive brain network, which is modulated by the willingness to pay.	•Ventromedial prefrontal cortex (reward). •Functional connectivity to ventromedial prefrontal cortex, including amygdala, hippocampus, ventral stratum (computation of subject value). •Insula (sensory characteristics of food).	Identify psychological processes, investigate neural correlates, and predict behavior		
Yokoyama et al. (2014), *NI*	What are the brain regions associated with the perception of social risk when purchase decisions are being made.	In a purchase intention task, ratings of social risk during purchase decision are associated with activity in the left anterior insula. In a social risk task, ratings of social risk are associated with activity in the left temporal parietal junction and medial prefrontal cortex.	•Left anterior insula (emotion-related network). •Left temporal parietal junction and medial prefrontal cortex (theory of mind).	Investigate neural correlates		
Cascio et al. (2015), *JMR*	How consumers make recommendation decisions in light of the preferences and opinions of others.	Decisions to update other-directed recommendations in response to peer opinions may unite brain systems associated with social influence and the concept of being a “successful idea salesperson.”	•Precuneus/posterior cingulate cortex, dorsal, dorsal anterior cingulate cortex, ventral striatum, anterior insula, orbitofrontal cortex/dorsomedial prefrontal cortex (social influence). •bilateral temporo-parietal junctions, medial prefrontal cortex (successful recommendations).	Identify psychological processes	Yes	
Chen et al. (2015), *JMR*	Whether consumers have an *a priori* network in their minds that reflects personality traits associated with brands and, if so, what cognitive processes are captured by such a network.	An *a priori* neural network is distributed widely across the brain and reflects personality traits associated with brands.	A wide range of brain areas including the anterior cingulate, middle cingulate, dorsomedial prefrontal, medial prefrontal, premotor, dorsolateral prefrontal, posterior cingulate, primary visual, lateral prefrontal, and inferior frontal cortices; the insula; and the hippocampus.	Investigate neural correlates	Yes	
Genevsky and Knutson (2015), *PS*	What factors influence the outcomes of online microlending, and whether a neural activity may forecast this outcome.	Positive affect, both self-reported and captured by the activity in the nucleus accumbens can promote the success of loan requests.	Nucleus accumbens (positive arousal)	Identify psychological processes and predict behavior		Yes
Karmarkar et al. (2015), *JMR*	Whether seeing the price of a product before or after seeing the product influences the way the product is processed.	Price primacy (i.e., seeing the product price before seeing the product) makes consumers evaluate the product based on its monetary worth, whereas product primacy (i.e., seeing the product before seeing the product price) makes consumers evaluate the product based on its attractiveness and likability.	•Medial prefrontal cortex (perceived monetary value). •Nucleus accumbens (attractiveness or expressed preference).	Identify psychological processes		Yes
Plassmann and Weber (2015), *JMR*	What individual differences can predict the consumer responsiveness to the “marketing placebo effects” (i.e., the effect by which consumption experience and subsequent behavior are influenced by marketing-based expectations such as price, quality beliefs, etc.).	Reward seeking, somatosensory awareness, and need for cognition can predict marketing placebo effects.	•Gray matter volume of the striatum (reward responsiveness/learning). •Gray matter volume of prefrontal structures (i.e., lateral orbitofrontal, lateral prefrontal, and dorsomedial prefrontal cortex) (cognitive top-down processing). •Gray matter volume of the posterior insula and somatosensory cortices (Somatosensory bottom-up processing).	Identify psychological processes	Yes	Yes
Venkatraman et al. (2015), *JMR*	How to predict ads’ effectiveness using fMRI.	Product desirability measured at the brain level is associated with real-world, market-level response to advertising.	•Amygdala (affective processing). •Dorsolateral prefrontal cortex (cognitive processing). •Ventromedial prefrontal cortex, striatum (desirability).	Predict behavior		Yes
Falk et al. (2016), *SCAN*	Whether and the brain activation acquired in a small group of smokers react to an anti-smoking email campaign can predict the effectiveness of the campaign on a population-level. If yes, via what mechanism.	Neural activity in the medial prefrontal cortex acquired in a small group of smokers is predictive of population-level responses to the campaign. Self-related processing is the neurocognitive mechanism that links neural and behavioral responses.	Medial prefrontal cortex (self-related processing).	Identify psychological processes and predict behavior	Yes	Yes
Kühn et al. (2016), *NI*	How to predict product sales in the real market using fMRI.	fMRI signal acquired in a small sample in eight brain regions that are associated with product decision-making predicts the real-world sales of products shown in the scanner.	•Nucleus accumbens, medial orbitofrontal cortex, amygdala, hippocampus, inferior frontal gyrus, and dorsomedial prefrontal cortex (a range of mental processes associated positively with sales). •Dorsolateral prefrontal cortex and insula (a range of mental processes associated negatively with sales).	Predict behavior		Yes
Reimann et al. (2016), *JACR*	Why offering consumers the choice between a full-sized food portion alone and a half-sized food portion paired with a small non-food premium (e.g., a small toy or the mere possibility of winning frequent flyer miles) can motivate smaller portion choice.	Food and the expectation of receiving a non-food premium activate a common area of the brain (the striatum), which is associated with reward, desire, and motivation.	Striatum (reward processing).	Identify neural correlates and psychological processes	Yes	Yes
Chung et al. (2017), *JN*	What is the underlying mental process of decoy effect.	Underlying decoy effect seems to be context-dependent valuation. Moreover, deliberate control that monitors and corrects decision-making errors and biases is associated with the successful overcome of decoy effect.	•Left ventral striatum (reward value). •Right inferior frontal gyrus (response inhibition).	Identify psychological processes		
Genevsky et al. (2017), *JN*	Whether and, if yes, what neural activities can predict real crowdfunding outcomes weeks later from the time of fMRI scan.	The nucleus accumbens and medial prefrontal cortex both predict individual choices to fund in crowdfunding. However, only the nucleus accumbens predicts the market-level funding outcome.	•Nucleus accumbens (positive arousal). •Medial prefrontal cortex (value integration).	Predict behavior	Yes	Yes
Dal Mas and Wittmann (2017), *Cortex*	How consumers react to boredom and what are the neural systems underlying these reactions.	Consumer accept higher prices to avoid expected boredom, a behavioral bias modulated by caudate nucleus. During the actual performance of a boring task, insula is activated and is associated with individual differences in boredom-related decision making	•Caudate nucleus (reward). •Insula (experiencing boredom).	Investigate neural correlates		
Chan et al. (2018), *JMR*	How to profile brand image using neuroimaging methods.	Perceived cobranding suitability, brand image strength can be used to profile brand image.	Occipital cortex, precuneus, posterior cingulate cortex, parahippocampal gyrus, and temporoparietal junction (areas associated with visual processing, episodic memory, self-awareness, and the default network)	Predict behavior		Yes
Reimann et al. (2018), *JACR*	Whether brand betrayal is an extreme form of brand dissatisfaction.	Brand dissatisfaction can be distinguished from brand betrayal.	Dorsolateral prefrontal cortex, angular gyrus, caudate tail (betrayal experience)	Identify psychological processes and distinguish multiple processes	Yes	Yes
Schmidt et al. (2018), *JN*	What underlies the individual differences in making healthy food choices.	The gray matter volume in the ventromedial prefrontal cortex and the dorsolateral prefrontal cortex predict individual differences in shifting to healthier food.	Ventromedial prefrontal cortex and dorsolateral prefrontal cortex (reward and valuation)	Predict behavior		
Chan et al. (2019), *NI*	Whether the predictive power of neural similarity distributes across the whole brain or only in specific regions (“neural similarity” here refers to the similar neural responses to stimuli across individuals). Whether similarity acquired in a small group of individuals is known to predict preference for the stimuli in a larger population.	Neural similarity of temporal lobe and cerebellum across a small group of individuals can predict the preference and recall of stimuli (e.g., commercials or products) in a larger sample.	Temporal lobe and cerebellum (level of engagement with video stimuli, sensory integration, emotional processing).	Investigate neural correlates and predict behavior	Yes	Yes
Doré et al. (2019), *JN*	How affective reactivity, valuation, and emotion regulation interact to deliver the impact of emotionally evocative messages (e.g., ads).	Increased activation in the amygdala predicts the effectiveness of emotionally evocative message both on individual and population levels. This effect is mediated by activity in the ventromedial prefrontal cortex and moderated by an emotion regulation pattern in the brain.	•Amygdala (affective reactivity). •Ventromedial prefrontal cortex (integrative valuation). •Whole-brain pattern of emotion regulation.	Identify psychological processes and predict behavior		
Huang and Yu (2019), *NI*	What are the neural substrates of the money illusion (the phenomenon wherein consumers evaluate money based on its face value instead of its true purchasing power), in both win and loss domains.	Posterior insula encodes the true value of money in the win domain but not in the loss domain. The ventral striatum, ventromedial prefrontal cortex, and amygdala process money illusion in both win and loss domains. Functional connectivity is also associated with money illusion, such that individuals with greater money illusion exhibit stronger functional connectivity between the ventral striatum and ventral anterior cingulate cortex in the win domain, but stronger functional connectivity between the ventral striatum and amygdala in the loss domain.	•Posterior insula (emotional and somatosensory arousal). •Ventral striatum, ventromedial prefrontal cortex, ventral anterior cingulate cortex (reward). •Amygdala (emotion).	Investigate neural correlates		
Huijsmans et al. (2019), *PNAS*	How the “scarcity mindset” created by insufficient resources (e.g., state of poverty) influences consumer decision-making.	A scarcity mindset is associated with increased activity in the orbitofrontal cortex and decreased activity in the dorsolateral prefrontal cortex	•Orbitofrontal cortex (valuation). •Dorsolateral prefrontal cortex (goal-directed choice).	Identify psychological processes		
Warren and Reimann (2019), *JACR*	What distinguishes products that look cool from those that look funny.	Cool [humorous] products diverge from the norm in a way that does [does not] make sense. Thus, cool (vs. humorous) products are processed differently in the brain.	Anterior cingulate cortex (resolution of conflict in the environment, error detection, and problem resolution).	Identify psychological processes and distinguish multiple processes	Yes	Yes
Wiggin et al. (2019), *JCR*	How curiosity influences indulgence behavior.	Curiosity gives rise to desire for rewards, which in turn encourages indulgent behavior.	Insula (desire for rewards)	Identify neural correlates and psychological processes		Yes
Tong et al. (2020), *PNAS*	What are the neural mechanism underlying online video watching.	Video viewing engagement (duration, frequency, etc.) can be predicted by increased activity in the nucleus accumbens and medial prefrontal cortex, and decreased activity in the anterior insula.	•Nucleus accumbens (positive arousal). •Medial prefrontal cortex (value integration of affect with other considerations). •Anterior insula (negative or general arousal).	Investigate neural correlates and predict behavior		

*JACR, *Journal of Association for Consumer Research; JAMS*, *Journal of the Academy of Marketing Science; JCR*, *Journal of Consumer Research; JMR*, *Journal of Marketing Research; JN*, *Journal of Neuroscience; JNPE*, *Journal of Neuroscience, Psychology, and Economics; NI*, *NeruoImage; PNAS*, *Proceedings of the National Academy of Sciences of the United States of America; PS*, *Psychological Science; SCAN*, *Social Cognitive and Affective Neuroscience.*

## Approaches for the Interpretation of Consumer Neuroscience Data: Forward Versus Reverse Inference

According to Wixted and Mickes (2013), neuroimaging studies take at least two major interpretative approaches. The first approach examines the neuroanatomical localization of behaviors and mental tasks, a process often referred to as *forward inference* (Henson, 2005; Poldrack, 2011). Forward inference has been adopted in consumer neuroscience to investigate the neurophysiological substrates of a wide range of consumer research concepts including, but not limited to, valuation (De Martino et al., 2009), social influence of buying decisions (Yokoyama et al., 2014), perception of money (Huang and Yu, 2019), and consumer curiosity (Wiggin et al., 2019). A typical forward inference question is this: While the concept of consumers’ willingness to pay (WTP) for a product is not unfamiliar to consumer researchers, where is WTP encoded in the brain? Plassmann et al. (2007) attempted to answer this interesting question by having hungry participants bid for the right to eat food. The authors pinpointed the medial prefrontal cortex and the dorsolateral prefrontal cortex as encoding locations of WTP. While the forward inference approach is laudable, it does not come without limitations. One such limitation is that while forward inference may highlight that a particular consumer research concept is correlated with activation of a certain brain area, forward inference often leaves open the psychological interpretation of possible underlying processes at play. This limitation may be less problematic in research that is largely interested in brain mapping but becomes more challenging in research that also cares about underlying psychological processes.

The second major approach of neuroimaging research involves using brain activation patterns to infer psychological processes. Specifically, by building on previously established functions and/or connectivity of specific brain regions, consumer researchers can make educated guesses as to which cognitive and emotional processes may be taking place as consumers process information or make decisions. This process is known as *reverse inference* (Poldrack, 2006, 2011). For instance, if a researcher posits that visual processing is taking place in an fMRI study because of increased activation in the occipital lobe, the researcher is reverse inferencing.

Reverse inference is important for consumer research (and for neuroscience in general) because it allows consumer researchers to access implicit or latent physiological processes (Reimann et al., 2011; Karmarkar and Plassmann, 2019). Specifically, the reverse inference approach can contribute to consumer research in at least three ways, as discussed below.

The first and most direct application of this research approach is that it allows consumer researchers to allude to underlying implicit psychological processes by observing activities of specific brain regions. This application requires a previously established link between brain regions and their functions. For instance, in studying how consumers process aesthetic package design, Reimann et al. (2010) recorded participants’ brain activity as they viewed aesthetic (vs. standardized) product package designs using fMRI. The authors observed that when processing aesthetic (vs. standardized) package designs, participants’ brains featured greater activation in the striatum, a brain area that has been associated with reward evaluations and processing (Delgado et al., 2003; Balleine et al., 2007; Kable and Glimcher, 2007; Báez-Mendoza and Schultz, 2013), among other activations. Following a similar interpretative logic, Plassmann et al. (2008) found that increasing the price of wine increases both reports of pleasantness as well as activity of the medial orbitofrontal cortex, a brain area that has widely been thought to encode pleasantness.

Second, neuroimaging can aid consumer researchers when competing theories exist to explain an observed phenomenon. Specifically, researchers have relied on established theories of brain regions’ functions to determine which theory is (or theories are) supported by actual brain activation. One such paper is Hedgcock et al. (2012) seminal examination of self-control depletion. Building on a two-stage model of self-control (i.e., recognizing the need for self-control → implementing self-control), the authors examined three completing models of depletion: (1) depletion only impairs the ability to recognize the need for self-control, (2) depletion only impairs the ability to implement self-control, and (3) depletion impairs both abilities. An fMRI study demonstrated that under depletion, a brain region associated with the implementation of controlled action (i.e., the right middle frontal gyrus located in the dorsolateral prefrontal cortex) features reduced activation, but such patterns were not found in the brain region associated with the detection of goal conflict (i.e., the anterior cingulate cortex). Therefore, the authors were able to conclude that only the implementation stage was likely to be affected by depletion (Hedgcock et al., 2012).

Third, the reverse inference approach is also useful in distinguishing different processes, networks, or stages of processing (i.e., neurophysiological dissociation). Such studies usually involve the observation of activations in different brain functional regions and/or networks (and occasionally also at different times) when participants undertake different cognitive tasks. For instance, Yoon et al. (2006) investigated whether semantic judgments of brands and humans are processed similarly. It has long been observed that consumers, marketers, and researchers alike tend to use similar if not identical words to describe both brands and people (Fournier, 1998; Fournier and Alvarez, 2012; MacInnis and Folkes, 2017). For instance, a brand can be “reliable” like a person and can form relationships with humans as other humans do. This tendency naturally raises the question of whether brands and humans are processed identically or at least similarly in the human brain. Yoon et al. (2006) work, however, showed that this may not be the case: Processing humans was associated with the medial prefrontal cortex, whereas processing brands was associated with the left inferior prefrontal cortex, which has been found to be involved in object processing.

In summary, neuroimaging studies often take two major interpretative approaches: Using both behaviors and mental tasks to explore neurophysiological correlates and to map the brain (i.e., forward inference) and using brain activations to infer certain psychological processes (i.e., reverse inference). With reverse inference, we have further identified three ways in which reverse inference contributes to consumer research: (1) determining underlying, implicit, and unknown psychological processes; (2) determining the plausibility of enhancing and/or completing theories; and (3) distinguishing multiple processes, networks, or stages of processing. Both approaches are of great value and are widely employed in consumer neuroscience and neuroscience in general. In the present paper, we focus on reverse inference due to its theoretical and methodological intricacies and highlight its potential issues when employed.

## Potential Issues Associated With Reverse Inference

Although reverse inference is by no means an incorrect research approach *per se* (Hutzler, 2014), neuroscientists have noticed interpretational issues associated with it (Poldrack, 2006; Del Pinal and Nathan, 2017). More specifically, when activation in a brain region of interest is not particularly selective for a specific cognitive process (i.e., when multiple cognitive processes can activate the same brain region), the validity of reverse inference can be undermined (Poldrack, 2006, 2011).

For instance, it has long been known that the anterior cingulate cortex is heavily involved in the processing of conflicting information from the environment (Botvinick et al., 2001; Van Veen et al., 2001; Carter and van Veen, 2007), so it is tempting to assume that the consumer brain is processing conflicting information when activation in this brain region is observed. However, prior literature shows that the anterior cingulate cortex is also sensitive to the gain and loss of rewards (e.g., Taylor et al., 2006; Seo and Lee, 2007). As a result, researchers cannot, without committing a logical fallacy, argue for the involvement of conflicting information based on the activation observed in the anterior cingulate cortex alone.^1^

Unfortunately, multi-functional brain regions are quite common (e.g., Miller and Cohen, 2001; Postuma and Dagher, 2006). As a result, issues associated with reverse inference are neither foreign to consumer neuroscience nor foreign to neuroscience in general. Indeed, in the recent consumer neuroscience literature, the medial prefrontal cortex has been associated with reward processing (Reimann et al., 2010; Karmarkar et al., 2015), pleasantness (Plassmann et al., 2008), self-referential evaluation of preferences (Hedgcock and Rao, 2009), and social processes (Dietvorst et al., 2009; Cascio et al., 2015). While the exact three-dimensional locations of the medial prefrontal cortex may slightly vary across these investigations, they generally belong to the same anatomical brain area. Similarly, activation in the insula has been claimed to indicate negative affect status (Berns et al., 2010), social influence (Cascio et al., 2015), and desire for rewards (Wiggin et al., 2019).

What, then, can and should consumer researchers do to address this issue? Below, we will focus on three major strategies that can mitigate potential issues inherent in reverse inference: (1) reducing false alarms via refined study design and analysis, (2) employing neuroimaging meta-analyses to estimate the extent of reverse inference, and (3) integrating neuroimaging data with psychometric assessments and data from behavioral studies to provide additional confidence in the findings. We also posit that data triangulation employing these methods may greatly enhance the validity of neuroimaging research.

## Addressing Potential Issues Associated With Reverse Inference

To address the potential issues associated with reverse inference, we first explain its logic. Let *COG* be the cognitive process of interest, and let *ACT* be the activation in the brain region purportedly associated with this process. The validity of reverse inference is thus given by the probability that *COG* takes place given that *ACT* is present, or, formally, *P*(*COG*|*ACT*). According to Bayes’ theorem:

$$P\,\left(C\,O\,G|A\,C\,T\right)=\frac{P\,\left(A\,C\,T|C\,O\,G\right)\,P\,\left(C\,O\,G\right)}{P\,\left(A\,C\,T\right)}$$

It should be noted that the term *COG* here is always conditioned on the specific task used in the studies. In other words, the prior probability of *P*(*COG*) in this equation should in fact be P(*COG*|*TASK*), where *TASK* stands for the task setting and design. We have omitted this term in the equation for the sake of succinctness, but we will revisit this task-relevant nature of reverse inference in later discussions.

The equation above translates the question of reverse inference into “When the activation of specific brain regions is observed, how likely is it that the cognitive process of interest indeed took place?” To be able to make any meaningful argument based on such reverse inference, consumer researchers need to estimate and, if possible, systematically increase the value of *P*(*COG*|*ACT*). This can be done in multiple ways, as summarized below.

### Reducing False Alarms in Reverse Inference via Careful Design and Analysis

Activations in a brain region that (1) is purportedly associated with a cognitive process of interest but (2) does not actually reflect the proposed cognitive are called *false alarms*, which is a major culprit of reverse inference’s validity issues (Hutzler, 2014). Mathematically, the denominator in the above equation, *P*(*ACT*), is the prior probability that indicates the tendency of this brain region to become activated *by default*. If a brain region can easily become activated (i.e., if the false alarm rate is high) – because, for instance, it is involved in many different cognitive processes – then *P*(*COG*|*ACT*)will be relevantly small because *P*(*ACT*) is large, rendering reverse inference invalid.

Although how easily a brain region can become active and in how many different functions a brain region can get involved are questions largely beyond consumer researchers’ control, methods exist to control the level of *P*(*ACT*).

First, consumer researchers could design studies in such a way that the chances of false alarms are reduced. To illustrate, the term *P*(*ACT*) in the equation of Bayes’ Theorem can be further broken down to *P*(*ACT*|*COG*)*P*(*COG*) + *P*(*ACT*|¬*COG*)*P*(¬*COG*); thus,

$$P\,\left(C\,O\,G|A\,C\,T\right)=\frac{P\,\left(A\,C\,T|C\,O\,G\right)\,P\,\left(C\,O\,G\right)}{\begin{array}{c} P\,\left(A\,C\,T|C\,O\,G\right)\,P\,\left(C\,O\,G\right)\\ +P\,\left(A\,C\,T|\neg \,C\,O\,G\right)\,P\,\left(\neg \,C\,O\,G\right) \end{array}}$$

where ¬*COG* indicates that the cognitive process of interest is not present.

According to this equation, if a brain region can be activated by a mental process different from the one of interest (i.e., the false alarm, the probability of which is captured by *P*[*ACT*|¬*COG*]), then the validity of reverse inference *P*(*COG*|*ACT*) will decrease as a result (all else being equal). Conversely, if, based on the systematic review of prior literature and study design, consumer researchers can lessen the possibility that such alternative cognitive processes take place, then the term *P*(*ACT*|¬*COG*)*P*(¬*COG*)] should shrink in value, and, as a result, the validity of reverse inference will increase. For instance, in a thought experiment, Hutzler (2014) discusses an experiment that infers the recognition of visual words from activation of the fusiform gyrus. Because the imagery study only involves the presentation of words, consumer researchers can reasonably rule out the competing cognitive theory of facial recognition. In this way, although the fusiform gyrus is multifunctional, the study design creates functional specificity by eliminating an alternative theory and, thus, controlling the baseline level of *P*(*ACT*), which is in fact *P*(*ACT*|*TASK*). Needless to say, ruling out alternative cognitive processes via design requires a good understanding of the literature on the brain region(s) of interest and depends heavily upon how much is known about the functionality of the brain region (cf. Agis and Hillis, 2017).

Another way to reduce false alarms is to use finer (i.e., smaller and/or better-defined) structures for reverse inference. Smaller ROIs are more selective and, thus, more predictive of cognitive processes than larger regions (Poldrack, 2006). In addition, instead of relying on the activation of individual brain regions, consumer researchers could use patterns of such activations to decode underlying cognitive processes, a method called multi-voxel pattern analysis (MVPA). This method utilizes pattern recognition to identify connections between cognitive processes and neuroimaging activation patterns. Such connections can then be used to reduce false alarms, as holistic activation patterns are much more selective for specific cognitive processes than individual brain regions are (Norman et al., 2006; Poldrack, 2008, 2011; Mahmoudi et al., 2012; Del Pinal and Nathan, 2017).

### Integrating Information From Neuroimaging Meta-Analyses

Why can meta-analyses help validate reverse inference? To answer this question, we must first discuss two important notions that are pertinent to reverse inference: consistency and specificity (Wager et al., 2009). *Consistency* is the extent to which brain activations replicate across studies, scanners, and labs, when certain cognitive processes are engaged. For instance, when participants see fear-inducing stimuli in a scanner, do their amygdalae tend to become activated regardless of where and in which labs they are scanned, who is scanning them, and how fear is induced in these scans? If the answer is “yes,” then the amygdala can be assumed to be consistently associated with fear. In the equation above, consistency is captured by *P*(*ACT*|*COG*), which is in proportion to *P*(*COG*|*ACT*). In other words, if consistency is inflated, then the evaluation of reverse inference will also be inflated, causing a validity issue. Unfortunately, in neuroimaging studies, *P*(*ACT*|*COG*) does tend to be inflated, because the usually small sample sizes and large numbers of tests give rise to underpowered studies and high false positive rates (Wager et al., 2009; Yarkoni et al., 2011). As a result, to evaluate the validity of reverse inference, consistency must be accounted for. On the other hand, *specificity* captures whether a brain region is selective for a mental process, which is directly associated with the validity of reverse inference as discussed in section “Reducing False Alarms in Reverse Inference via Careful Design and Analysis.”

Therefore, to estimate *P*(*COG*|*ACT*) and assess the validity of reverse inference, it is crucial to estimate consistency and specificity. However, neither feature can be acquired from a single study. Rather, one must consult a substantial number of studies to make a good estimation of consistency and specificity and, in turn, the validity of reverse inference.

Neuroimaging databases, such as neurosynth.org (Yarkoni et al., 2011), attempt to provide a means to account for consistency and specificity based on prior neuroimaging literature and meta-analyses generated using the database. Such databases thus attempt to quantify the extent of reverse inference by estimating the posterior probability *P*(*COG*|*ACT*). Neurosynth.org claims to be an open-source, large-scale, automated synthesis of functional neuroimaging data, utilizing text-mining and meta-analytic techniques to synthesize more than 14,000 published research papers (as of June 2020) and providing probabilistic mappings between brain regions and terms. These terms describe cognitive states (e.g., “pain,” “working memory,” or “fear”) and are used in the abstract of a paper, so they serve as a proxy of the mental process of interest. Based on this database and the meta-analysis provided therein, consumer researchers are able to tell whether there tends to be a non-zero association between the usages of certain terms (e.g., “emotion”) and the activation of a given brain region (e.g., the amygdala) in the extant literature. More importantly, the database also provides the posterior probability of a term, if the activation of a specific brain region is found (i.e., an estimated *P*(*COG*|*ACT*) based on extant literature).

To illustrate the usefulness and usage of meta-analysis, imagine that a consumer researcher is interested in how product evaluation can be influenced by the physical attractiveness of photos of human models that appear on product packages. Let us further assume after a literature review, the researcher hypothesizes that highly attractive models are more rewarding than average-looking models and thus lead to more favorable product evaluation. Since the reward valuation of physical attractiveness is associated with activity in the ventromedial prefrontal cortex (O’Doherty et al., 2003; Pegors et al., 2015), the researcher chooses the ventromedial prefrontal cortex as the ROI that reflects reward processing in this hypothesis. [Note, however, that in this scenario we focus on a single brain region only to make the example as simple as possible. In reality, it is always advisable to expect more than one ROI to be activated and, as a result, to (1) always include a whole brain analyses under the proper family-wise error rate (FWER)/false discovery rate (FDR) threshold (i.e., techniques to control false positives in fMRI data analysis, see Bennett et al., 2009) and/or (2) investigate brain regions in their activation patterns rather than in isolation, for instance employing MVPA as discussed in Section “Reducing False Alarms in Reverse Inference via Careful Design and Analysis”].

The researcher then sets out to test the hypothesis with fMRI, in which participants are shown a battery of product packages with images of either highly attractive models or average-looking models on them. Consistent with the hypothesis, when viewing packages with highly attractive (vs. average-looking) models, participants exhibit a brain activation in the ventromedial prefrontal cortex, in a region of interest with the center being the MNI coordinates *x* = −4, *y* = 38, *z* = −16. Also consistent with the hypothesis, the BOLD signal in the ventromedial prefrontal cortex mediates the liking of the product reported by in-scanner button pressing.

Based on these results, it would seem that the hypothesis is supported by the fMRI result. However, is this truly the case? Can the researcher reasonably infer reward processing from this brain activation, understanding the risks of reverse inference, especially regarding such notoriously multifunctional regions as the ventromedial prefrontal cortex?

**FIGURE 1:**
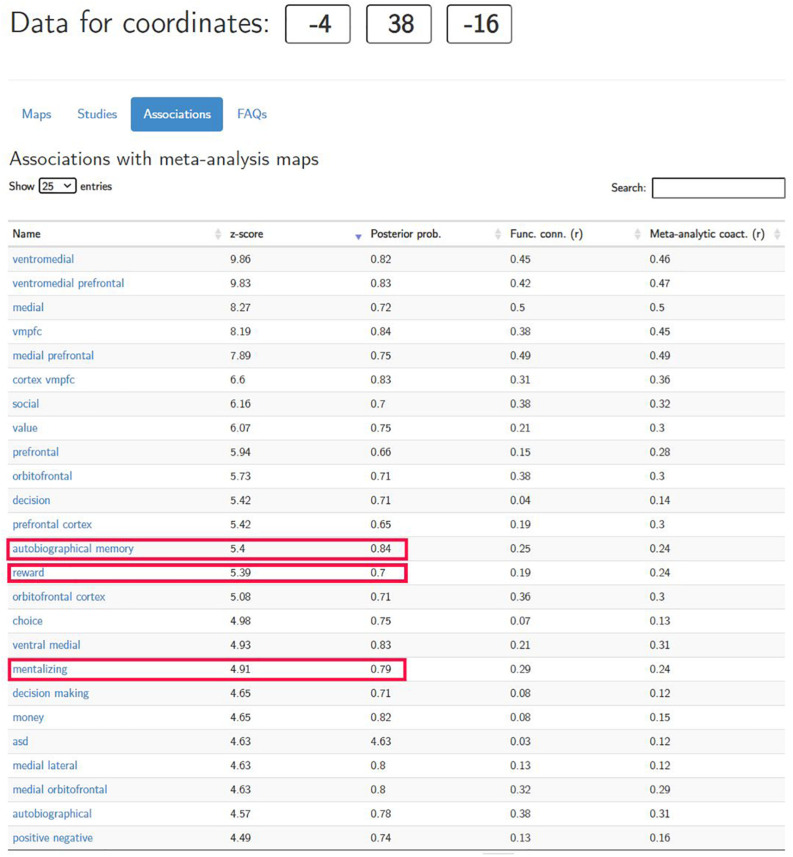
Meta-analysis results generated by neurosynth.org (retrieved on June 26, 2020).

To answer this question, the researcher needs to estimate *P*(*RewardProcessing*|*vmPFCactivationinandaround*[−4,38,−16]), which indicates how much confidence the researcher can put in this reverse inference. Neurosynth.org is designed precisely to quantify such reverse inferences. After inputting the coordinates of the peak voxel activation in neurosynth.org, it generates a table under the “Associations” tab (see Figure 1) based on its meta-analytical data corpus. Clearly, some terms in the table are brain regions (e.g., “ventromedial,” “vmpfc”), while others are cognitive processes (e.g., “terms,” “decision,” “choice”).

Not surprisingly, the term “reward” (highlighted in Figure 1) appears in the association table. For this term of interest, the meta-analysis first returns a *z*-score of 5.39. This z-score shows whether there exists a non-zero relationship between the term (“reward” in this case) and the location (i.e., *x* = −4, *y* = 38, *z* = −16). The larger the z-score, the more confidence the researcher can have in claiming that this brain location is indeed associated with reward in the extant literature. Note that this z-score, however, *cannot* be used to determine the validity of the reverse inference being employed, *nor* can it be used to infer the strength of the association between the mental process (i.e., the term) and the location of the activation in the brain (Yarkoni, 2015b).

More informative is the number under the “posterior prob.” column. This number is the estimation of *P*(*COG*|*ACT*) and, thus, of the validity of the reverse inference. In our case, the posterior probability is 0.7 (see Figure 1). However, what does this number mean? Note that Bayes’ Theorem dictates that posterior probabilities must be calculated based on prior probabilities. In the case of neurosynth.org, its creators arbitrarily impose a uniform 50% prior probability on *all* terms [*P*(*COG*) =  0.5*foranyCOG*]. In other words, neurosynth.org assumes that if we take a random paper in the database, the prior probability of that paper using *any* term is exactly 50%. This is, of course, not true in reality; however, by making this assumption, neurosynth.org provides a threshold that applies to *all* terms, such that if the posterior probability is greater than 0.5, then the researcher can reasonably assume that the reverse inference he or she is examining has some merit at an above-chance level (Yarkoni et al., 2011; Yarkoni, 2015b). In addition, this uniform, arbitrary 0.5 prior probability allows researchers to make quantitative comparisons across all terms, such that if a location has a greater posterior probability for a term, then this region is more preferentially associated with that term than are the terms with lower posterior probabilities. It should be noted, however, that if the arbitrary prior probability is changed to other values from 0.5, the posterior probability will also vary as a result. Therefore, the absolute value of the posterior probability does not carry much meaning *per se*, and interpretations thereof should be made only in relation to other probabilities, such as the default 0.5, or the posterior probability for a different term associated with the same voxel.

Therefore, in our case, the 0.7 posterior probability means that if there is a 50–50 chance that a study uses the term “reward” *a priori*, then the estimated posterior probability for the term “reward” to be used increases to 70% after we observe the activation of the ventromedial prefrontal cortex in and around MNI coordinates *x* = −4, *y* = 38, *z* = −16. Thus, it seems that there is an above-chance likelihood that reward process is present in this hypothetical study. Please note again that one can only interpret change (i.e., 0.7 vs. the baseline 0.5) and not the absolute value.

However, we should take care here to avoid jumping to conclusions. It is very important to note that the fact that there is a decent chance of reward processing having taken place given the observed activation does not necessarily mean that this region is *selective* for reward. Rather, other terms may also have comparable or even higher levels of posterior probability in this reported region. Indeed, if we pay close attention to other terms in Figure 1, terms such as “autobiographical memory” and “mentalizing” (both highlighted) will arise, each having a posterior probability even greater than that of “reward” (0.84 and 0.79, respectively). Admittedly, many terms with the highest posterior probabilities in this table are reward-related (e.g., “reward,” “value,” “money”). However, even after taking all these terms into consideration, the conclusion the researcher can draw from this meta-analysis is still inconclusive; that is, the ventromedial prefrontal cortex in and around (*x* = −4, *y* = 38, *z* = −16) is preferentially (but not selectively) associated with reward. In other words, at this point in the analysis, we are still not sure if reward processing is truly happening.

Thus far, we have discussed what the neurosynth.org database tells us and, equally importantly, what it does *not* tell us (also cf. Yarkoni, 2015a,b for a discussion). The most important question, however, still remains. The researcher in our hypothetical example theorizes that reward is underlying the processing of product packages with images of highly attractive (vs. average-looking) human models on them. The fMRI result seems to support this argument, and the meta-analysis suggests that there is a good chance that the researcher is on the right track. However, the researcher still cannot draw decisive conclusions, due to the existence of alternative, equally viable (at least judging from the meta-analysis) mental processes (in this case, autobiographical memory and mentalizing). So, what should the researcher do now?

This question can be partially addressed by the meta-analysis that brings up this very question. Indeed, a valuable function of meta-analysis is to identify a (limited) set of competing theories so the proposed hypothesis can be supported by eliminating other (at least major) alternatives. Needless to say, the easiest alternatives to exclude are those that are unlikely to occur under the particular study design being employed. For instance, in the design of the hypothetical study described above, autobiographical memory is very unlikely to have been involved (since there is not a memory-related task involved) and thus can be reasonably excluded.

However, mentalizing – the process by which individuals make sense of the mental state of others or oneself – may well survive this “exclusion by study design.” After all, when consumers see other human beings (i.e., human models used in our hypothetical fMRI experiment), especially if they show emotions, consumers may well engage in mentalizing and, in turn, feature the well-documented activation in the ventromedial prefrontal cortex (Lombardo et al., 2010; Atique et al., 2011; Schnell et al., 2011). At this point, the researcher needs to think above and beyond the meta-analysis. One possible solution, for example, is to acquire the activation pattern of mentalizing; if this pattern is inconsistent with the actual results, then it is very likely that mentalizing may not have taken place. In this case, mentalizing involves not only the activation of the ventromedial prefrontal cortex but also activity in the precuneus and/or the temporoparietal junction, which often accompany ventromedial activations. Thus, if the researcher fails to find meaningful activations in these additional regions, mentalizing may not have been engaged in the study. Needless to say, the researcher should turn to extant literature to identify such patterns; however, another newly launched meta-analysis database, neuroquery.org, may also greatly assist in this process, in that it helps researchers predict which brain regions are likely to become activated given designated cognitive processes. Moreover, pattern-based activation can prove useful even if mentalizing does take place. For instance, if reward processing indeed occurs, it may not only involve the ventromedial prefrontal cortex but also trigger the orbitofrontal cortex and the ventral striatum. If the researcher can find activity in these regions that are also involved in reward processing, it becomes more evident that reward is taking place, even in the presence of mentalizing.

Although meta-analyses can shed important light upon the validity of reverse inference, it is not without limitations. Other than the information meta-analysis cannot yield, which we already discussed in Section “Integrating Information from Neuroimaging Meta-Analyses,” there exist other factors that limit the usefulness of meta-analyses.

First, when the term related to the cognitive process of interest has been rarely examined in the literature, the database will provide little information to the researcher. For instance, consumer researchers interested in personal control may recognize the scantiness of neuroimaging studies investigating perceived control and loss thereof, despite the richness of non-imaging work, which has been conducted mostly on non-human animals (e.g., Maier and Watkins, 2005; Amat et al., 2010, 2014).

Second, when a term is used inconsistently in the literature, the usefulness of meta-analysis is also limited. For instance, in the literature, the term “control” not only is used to refer to “personal control” but also is found in works involving self-control, motor control, executive control, and even the control condition in experimental studies. These terms are not distinguishable in the database due to the limitation of text-recognition tools, thus rendering meta-analyses on such terms virtually uninformative.

Third, meta-analysis databases like neurosynth.org cannot capture nuances across studies and designs. Such situations may introduce noise to meta-analyses and thus undermine the usefulness of their results, except in the case of well-defined and consistently-used cognitive processes and experimental procedures (Dockès et al., 2020).

For instance, meta-analysis databases may not be able to distinguish between “perceptual curiosity” and “epistemic curiosity,” as the terms tend to be aggregated together, despite their conceptual and neurophysiological distinctions in the literature on curiosity (Loewenstein, 1994; Jepma et al., 2012; Kidd and Hayden, 2015; Wiggin et al., 2019). To make matters worse, there are too few neuroimaging studies on curiosity to provide sufficient statistical power for the meta-analysis, if such terms exist in the database at all. Moreover, the inconsistent study procedures introduce noise to the database. Thus, if a researcher hypothesizes perceptual (but not epistemic) curiosity as the cognitive process underlying a certain consumer behavior, meta-analysis might not be able to provide much useful insight. In the following section, we argue that behavioral data can prove useful in addressing these concerns.

In addition, another database, NeuroQuery.org (Dockès et al., 2020), has recently been made publicly available.^2^ This new database complements the previously discussed databases in important ways, which we discuss below. An issue with the traditional databases is that the results they generate are largely based on decontextualized, aggregated data and arbitrary thresholding. Specifically, such databases rely on automated text-analysis algorithms and scripts to analyze and document the neuroscience concepts (in the form of “terms”) included in a sizable corpus of literature with considerable statistic power. However, to investigate these neuroscience concepts, the original research papers included in the corpus may take different approaches, employ different designs, and use different terms. Due to current limitations of text-mining techniques, traditional databases are unable to capture such task-related information, nuances across studies and designs, and idiosyncratic usages of the same terms. This situation can prove problematic, because the output of such analysis can suffer from a low signal-to-noise ratio and thus can be difficult to control and interpret, except in the case of well-defined concepts and/or highly standardized procedures (Dockès et al., 2020). Neuroquery.org, on the other hand, attempts to employ a different approach. Specifically, NeuroQuery.org begins with keywords (e.g., psychological processes, neuropsychological conditions, and anatomy) and uses these terms to predict what brain regions are likely to become activated if proposed psychological processes are engaged. The model this database utilizes can produce accurate and usable predictions of ROIs even for less common terms, terms that are named inconsistently in neuroscience vocabulary, or sets of terms that have not been studied together previously.

### Integrating Psychometric and Behavioral Data

Although many researchers turn to neuroimaging techniques to discover mental processes precisely because of behavioral studies’ limitations, psychometric and behavioral data are useful in neuroimaging studies in many ways. To begin with, in relation to our discussions on meta-analyses, psychometric and behavioral data can prove helpful in compensating the shortcomings of meta-analyses, as they can provide a better-calibrated, task-relevant environment to investigate psychological processes of interest. In the aforementioned case of curiosity, for instance, extant literature provides rich behavioral manipulations to introduce perceptual vs. epistemic curiosity (e.g., Loewenstein, 1994), and the researcher can thus conduct behavioral experiments to observe whether perceptual (but not epistemic) curiosity produces the consumer behavior in question. If so, such studies can provide more evidence to complement the fMRI data.

Even when meta-analyses can be employed without the issues discussed in the previous section, psychometric and behavioral data can still prove helpful to reverse inference studies by provide additional evidence that the proposed cognitive process is indeed implicated in the phenomenon being studied. Mathematically, doing so increases *P*(*COG*) and in turn *P*(*COG*|*ACT*). For instance, Plassmann and Weber (2015) investigate the consumer responsiveness to the “marketing placebo effects” (MPE; the effect by which consumption experience and subsequent behavior are influenced by marketing-based expectancy such price, quality beliefs, etc.). The authors provide a comprehensive account of the cognitive processes that can influence MPE: reward responsiveness, cognitive top-down processing, and somatosensory bottom-up processing. In an MRI study, the gray matter volume of brain regions associated with these cognitive processes is found to be predictive of the MPE effect. To provide further support for this theoretical account, the authors further conducted a study demonstrating that personality traits related to these cognitive processes are also predictive of MPE, as are gray matter volume measures, thus fostering extra confidence in the proposed processes.

Note that for triangulation purposes, the behavioral data do not need to be “purely behavioral” (i.e., collected from separate studies that do not feature neuroimaging elements at all). Rather, the collection of psychometric and behavioral data can either be part of the original fMRI study and/or collected in separate studies.

For instance, imagine a study investigating how food print ads drive consumers’ willingness to purchase. To explore the cognitive process underlying food ads, suppose the researcher shows hungry participants multiple food ad images in the scanner as they report how much they want to purchase the food advertised. Let us further assume that the researcher finds that more successful ads are associated with (1) a neural network associated with reward and impulse (which might include, say, the nucleus accumbens, orbital prefrontal cortex, ventromedial prefrontal cortex, anterior insula, etc.) and (2) importantly, reduced activation in the medial prefrontal gyrus, a region associated with inhibition behavior (e.g., Batterink et al., 2010). Based on this pattern, our hypothetical researcher speculates that more successful food ads might be associated with greater inhibition/control failure and, in turn, higher willingness to purchase. (Here again, we caution our readers that the theorization and selection of ROIs herein are overly simplified for the sake of clarity and succinctness).

This theorizing is clearly an example of potentially problematic reverse inferences. How, then, can behavioral data help the researcher with this proposed process? Of course, the aforementioned “purely behavioral” studies can be conducted, wherein the researcher shows that depleted participants exhibit greater willingness to purchase to the same food ad than non-depleted participants do, thus demonstrating the involvement of control process. Alternatively, the researcher could show that people’s acceptance of the same food ad is a function of their trait self-control. However, the researchers can also collect behavioral data and combine them with neuroimaging data collected in either the existing or a new study to provide support for the proposed hypothesis.

For instance, if inhibition/control indeed plays a role in the acceptance of food ads, the aggregated activation in related brain regions acquired from a group as small as the participants scanned may be predictive of the effectiveness of the ads in the real world on a market or population level (i.e., neuroforecasting, Genevsky et al., 2017). Such “related brain regions” may be the medial prefrontal gyrus found in the existing hypothetical study or may involve a more extensive inhibitory network established by the literature. Similarly, the researcher could also run a structural scan to see whether the cortical thickness of these brain regions is predictive of individual participants’ acceptance of the ads (e.g., rating, choice, etc.). Furthermore, the researcher can even use transcranial magnetic stimulation (TMS) to temporarily disrupt the function in these brain regions (if they are relative shallow) or recruit participants with lesions in these areas and obverse how these participants react to the ads in comparison to control participants. In all these approaches, behavioral data are combined with brain data to shed light upon the underlying cognitive processes – in this case, inhibition and control.

As shown above, psychometric and behavioral data can be triangulated with brain data in a very flexible manner and can provide interesting and useful insights to researchers in many ways. Based on the specific issues they are investigating, the nature of the existing design and data, and the technology and resources available to them, researchers can select approaches to behavioral data to fit their needs.

### Summary: The Importance of Triangulation

In summary, in consumer neuroscience, researchers can address the validity issue of reverse inference by (1) refining the neuroimaging study design and analysis to reduce false alarms, (2) utilizing neuroimaging databases and the meta-analysis data therein to quantify the extent of reverse inference, and (3) using psychometric and behavioral data to provide additional process evidence.

In this paper, we advocate not the isolated employment but the triangulation of these methods. Such triangulation can enable each method to compensate for the others’ shortcomings and disadvantages, thus providing synergy toward improved overall validity. As discussed earlier, notwithstanding their unique advantages, each of these methods suffers from certain shortcomings: fMRI studies can investigate hidden mental processes but are potentially vulnerable to reverse inference issues; neuroimaging meta-analysis can assist researchers in quantifying reverse inference, but its usability can be limited and its analysis may not necessarily be conclusive; and behavioral data are flexible and can be well calibrated to the stimuli and procedures but may not be able to address the mental process of interest in a direct manner (the very reason researchers turn to neuroimaging in the first place).

Understanding the pros and cons of each method is important, as the cons serve as a starting point to identify the potential validity issues with one’s data, while the pros help researchers understand what data triangulations should be employed to address these issues. Indeed, this is precisely what the literature on Multitrait-Multimethod Matrices (MTMM, Campbell and Fiske, 1959; Bagozzi and Yi, 1993) suggests: that is, a single method may not be enough to distinguish the cognitive process of interest from unsubstantial, random-method variance and noise and, instead, multiple methods taking different angles can possibly redress single-method concern effectively.

Therefore, despite the increased time, effort, and expertise needed to adopt a multi-method approach, we still believe that the triangulation approach we have proposed herein should be considered by consumer neuroscience researchers. Specifically, for neuroimaging studies in consumer neuroscience, we believe the researcher should always (1) carefully refine the design of the fMRI study and (2) utilize meta-analyses (of course, sometimes the database is not usable for certain keywords and designs, but this is not known to the researcher until they have utilized the database). On the other hand, we believe that the researcher should base their decision of whether to include (3) psychometric and behavioral data, as well as what to include, on the specific issue they are facing. If, for example, the researcher makes a claim about certain mental cognitive processes based on brain activation, but the subsequent study design and meta-analysis cannot rule out competing theories and provide conclusive judgments, we would then recommend that psychometric and behavioral data be included. However, if it is already well-established in the behavioral literature that treatment X (e.g., seeing a picture of a snake) will cause mental activity M (e.g., fear) in the population of interest, and if, with due caution and prudence, the researcher can reasonably believe there is no good reason why this causal link would disappear in the scanner, then perhaps in such a case the researcher does not need to reinvent the wheel. Therefore, much as we applaud the merit of psychometric and behavioral data in general and believe they are beneficial most of the time, as a general principle we encourage researchers to include meaningful psychometric and behavioral data that address existing issues stemming from their particular research question and design, instead of triangulating for the sake of triangulating.

In addition to the hypothetical examples we used in previous sections, real-world examples of triangulation can also be found in the extant literature in consumer neuroscience. Regarding the works we summarized in Table 1, in Section “Integrating Psychometric and Behavioral Data,” we already discussed how Plassmann and Weber (2015) triangulated anatomical neuroimaging data with psychometric measures to explore the marketing placebo effect, and we would like to note that in this work the authors also utilize the NeuroSynth meta-analytical tool in support of their conclusions. Similarly, in Reimann et al. (2018), an fMRI study demonstrates that brand betrayal and brand satisfaction involve different brain areas. This result is triangulated with both psychometric measures and the NeuroSynth database. The authors were able to pinpoint a number of aspects on which brand dissatisfaction is differentiated from brand betrayal, including prior relationship with the brand, anger, and rumination. Furthermore, by combining fMRI, individual-level data and marketing-level behavioral data, Genevsky et al. (2017) found that nucleus accumbens activity not only predicts individual choices to fund in crowdfunding but also market-level funding outcome. The authors also examined the role sensory processes play in the identified pattern, and NeuroSynth was employed to predefine the volumes of interest (VOIs).

## Future Directions in Consumer Neuroscience

In addition to data triangulation, other practices can also improve the validity of individual studies and consumer neuroscience. Although a thorough discussion of these practices would be outside the scope of this paper, we would still like to take this opportunity to briefly advocate some of these practices for future studies in the field.

### Toward a Quantification of Triangulation

As discussed in Section “Summary: The Importance of Triangulation,” the notion of data triangulation proposed in this paper is consistent with that of the Multitrait-Multimethod Matrix (MTMM, Campbell and Fiske, 1959). The MTMM provides useful information that can benefit fMRI studies in three ways. First, the MTMM provides estimations of reliability of each measure, which could potentially help address the issue that fMRI measures tend to suffer from poor overall reliability (Elliott et al., 2020). Second, the MTMM provides assessment of convergent validity: if a concept (e.g., a mental process) is measured through multiple methods, the measures should be strongly correlated. Third, the MTMM also helps researchers to establish discriminant (or divergent) of measures, such that measures for different constructs should not be correlated (Campbell and Fiske, 1959).

Therefore, the MTMM would be a good tool to evaluate how well the triangulation improves the validity. The challenge is, however, how to directly compare the vastly different methods (e.g., behavioral data of distinct nature and neuroimaging data) on the MTMM. Although the analysis above already lays out the means in which triangulation can help improve the validity of fMRI studies, quantifying such improvement would provide the proposed triangulation with even more solid footing. Thus, we advocate the quantification of the proposed triangulation as a direction for future research.

### Toward an Interpretable Consumer Neuroscience

Recent years have witnessed an increase in the important discussion regarding how to promote openness and transparency of research in the fields of psychology and neuroscience (e.g., Aarts et al., 2015; Gorgolewski and Poldrack, 2016; Kidwell et al., 2016; Nichols et al., 2016; Gilmore et al., 2017; Klein et al., 2018; Shrout and Rodgers, 2018). In solidarity with the fields’ joint effort toward a more precise and interpretable discipline, we also take the opportunity to advocate these practices in consumer neuroscience. For example, we suggest that consumer researchers could report information regarding experimental design, image acquisition, preprocessing, statistical modeling and statistical inferences, as well as result tables, which could be provided in a web appendix alongside the main paper. Some guidelines and checklists are provided, for instance, by Nichols et al. (2016) and in the appendix to Reimann et al. (2018). These practices may be helpful to make research reports more interpretable.

Another laudable practice is data sharing. This practice is more than just academic integrity, but also improves the validity of data analysis. In a recent paper published in *Nature* (Botvinik-Nezer et al., 2020), 70 independent teams of researchers were asked to analyze the same dataset. Since no two teams employed the same workflow of analysis, this flexibility in data analysis creates a considerable variation in the results of hypotheses testing. When the results from teams are aggregated, however, they show a significant convergence on activated regions. Thus, for better cross-validation, consumer neuroscientists may want to consider sharing data, which can be done with dedicated online repositories such as OpenfMRI.org (Poldrack et al., 2013), NeuroVault.org (Gorgolewski et al., 2015), and OpenVoxel.org. Furthermore, we encourage consumer researchers to share the workflow by which the data were analyzed, and report (if applicable) the code they used to analyze scan data on online platforms such as GitHub.com to further promote transparency, openness, and cross-validation.

## Conclusion

Consumer neuroscience may hold valuable insights for the advancement of consumer research, with perhaps the most intriguing of these being its potential to (1) reveal hidden cognitive and emotional processes that have been inaccessible to traditional research methods and (2) to confirm physiological and psychological processes underlying consumer behavior. Indeed, over the years, consumer researchers have been employing fMRI, along with other neuroimaging techniques, in an attempt to open the black boxes of consumer experience, motivation, decision-making, and so on. Needless to say, fMRI has shed new light upon our understanding of consumer behavior, and we fully acknowledge the important contributions made by the field. That being said, as we pointed out earlier, many studies’ reliance on reverse inference may render them vulnerable to validity issues. To promote the validity of individual neuroimaging studies as well as consumer neuroscience as a field, in this paper we advocate for the triangulation of neuroimaging data with meta-analyses as well as psychometric and behavioral data. We believe these research practices may substantially increase the conclusions we can draw from fMRI data. Therefore, we encourage the employment of data triangulation in consumer neuroscience research. Lastly, we take this opportunity to offer our special acknowledgment to the intensive methodological discussions and disputes taking place in our parent disciplines, such as cognitive neuroscience and functional neuroimaging, as this paper draws largely upon their lessons and wisdom.

## Data Availability Statement

The original contributions generated for this study are included in the article/supplementary material, further inquiries can be directed to the corresponding author.

## Author Contributions

CC conducted the review on extant literature and the analysis on reverse inference in the frontend with the consent of MR. Both CC and MR drafted the frontend. Both authors contributed to the article and approved the submitted version.

## Conflict of Interest

The authors declare that the research was conducted in the absence of any commercial or financial relationships that could be construed as a potential conflict of interest.

## Footnotes

1 Note, however, that this issue related to reverse inference may not be present in studies in which researchers try to predict behaviors (e.g., choice and purchase) based on brain activation (i.e., “neuroforecasting”; for a review, see Knutson and Genevsky, 2018). This is because, in such prediction studies, the focus is to establish whether certain brain regions are predictive of actual behavior rather than to investigate the mental processes associated with such regions. For instance, if activations in the ventral striatum of the small number of individuals listening to music in a scanner are predictive of the market-scale sales of the music (Berns and Moore, 2012), then this prediction is valid in and of itself, and whether or not one can infer certain mental processes (e.g., reward processing) based on ventral striatum activation is irrelevant in such predictions.

2 The authors also thank an reviewer for bringing this new development to our attention during the review process.
